# Psychological distress during the first phase of the COVID-19 pandemic in Ecuador: Cross-sectional study

**DOI:** 10.1371/journal.pone.0257661

**Published:** 2021-09-22

**Authors:** Juan Gómez-Salgado, Ingrid Adanaque-Bravo, Mónica Ortega-Moreno, Regina Allande-Cussó, Cristian Arturo Arias-Ulloa, Carlos Ruiz-Frutos

**Affiliations:** 1 Department of Sociology, Social Work and Public Health, Faculty of Labour Sciences, University of Huelva, Huelva, Spain; 2 Safety and Health Postgraduate Programme, Universidad Espíritu Santo, Guayaquil, Ecuador; 3 Faculty of Engineering in Mechanics and Production Sciences, Escuela Superior Politécnica del Litoral, Guayaquil, Ecuador; 4 Department of Economy, Faculty of Labour Sciences, University of Huelva, Huelva, Spain; 5 Department of Nursing, University of Seville, Seville, Spain; Konkuk University, REPUBLIC OF KOREA

## Abstract

**Background:**

The effects of the COVID 19 pandemic on the mental health of citizens from Asia, Europe, or North America begin to be known, but there are fewer publications on its effects in Latin American countries. In this study, its impact in Ecuador is described, with data collected during the first phase of the pandemic. The objective of this study was to analyse the level of psychological distress in the population of Ecuador during the first phase of the COVID-19 pandemic.

**Methods and findings:**

Cross-sectional observational study. The questionnaires were collected through an online self-developed questionnaire, between April 2 and May 17, 2020, using the non-probabilistic sampling methodology: snowball method. The variables considered were sociodemographic variables, physical symptoms, health status, COVID-19 contact history, preventive measures, and the General Health Questionnaire (GHQ-12). The percentage with high psychological distress (PD) (GHQ-12 ≥ 3) has been somewhat lower than that found in Europe, being women, young people, people with higher level of education, living without a partner, not living with children or children under 16 years of age, and with worse perception of health the groups with the highest PD. Differences have been observed with European studies regarding common symptoms, preventive measures to avoid contagion, percentage of infected relatives, or diagnostic tests performed.

**Conclusions:**

The use of the same research instrument, validated in Europe and adapted to Ecuador, has facilitated the comparison of the found results and differences, which can be explained by socio-economic or cultural variables, the health system, level of information, or by preventive measures put in place to prevent the pandemic.

## Introduction

The SARS-CoV-2 virus disease (COVID-19) has caused a global health crisis with dramatic consequences. On 31 December 2019, the Wuhan Municipal Health Commission in China notified the World Health Organization (WHO) 27 cases of pneumonia of unknown origin [1]. On 30 January 2020, the WHO declared an international public health emergency following the COVID-19 outbreak that began in Wuhan, China. By that time, 83 cases had been identified in 18 different countries outside China [2, 3]. Following the increase in spread to more than 118,000 cases in 114 countries and 4291 deaths, on 11 March 2020, the WHO reported the pandemic status of the situation [4].

In Ecuador, the first confirmed case was reported on 29 February 2020 [5], and on 11 March, the Ministry of Health declared the State of Health Emergency in the National Health System (Agreement No. 00126–2020). Subsequently, restrictive measures were established on 16 March to prevent the spread of the virus, when the President of the Republic decreed the state of emergency (Executive Decree 1017), lasting 60 days [6]. The Committee on Emergency Operations (COE, for its acronym in Spanish) of the National Risk and Emergency Management Service, monitored compliance with quarantine and the set of measures that suspended the exercise of the right to freedom of movement, freedom of association and assembly, closure of the territory at the air, sea, and land level, implementing curfew, suspension of face-to-face working hours, and suspension of face-to-face classes in all levels nationwide. The state of emergency was renewed for 30 more days (Decree 1052), then for 60 (Decree 1074), and finally another 30 days (Decree 1126) [6], ending the state of alarm on September 13, 2020. The basic preventive measures proposed by the Emergency Operations Committee to prevent SARS-CoV-2 contagion were to keep a 2 metre-distance, cover mouth when sneezing, and to wash hands constantly [7].

The Ecuadorian National Emergency Operations Committee, together with the Decentralised Autonomous Governments (GAD, for its acronym in Spanish), coordinated the implementation of measures to prevent the spread of the virus and established protocols for economic reactivation [8]. In compliance with WHO’s recommendation to act against a variant of SARS CoV-2, which resulted in increased cases due to agglomerations, a new state of emergency was declared on 21 December, 2020 (Decree 1217) [6].

Cases had been reported in Ecuador and Brazil by February 2020, and also in early March in Chile, Colombia, and Peru [9]. In May, an increase in the number of confirmed infected cases and deaths in Brazil, Peru, Chile, Colombia, Argentina, Bolivia, Uruguay, and Paraguay [10] was evident. In September, the Latin American and Caribbean countries that had reported a higher number of confirmed cases and deaths per million inhabitants were Brazil, Peru, and Chile [9]. As of March 26, 2021, Ecuador officially reported 318,656 confirmed cases and 16,582 deaths, with 141,191 vaccine doses administered. Worldwide, Brazil was in third place at the contagion level and second in deaths, followed by Colombia (11^th^ in contagions and deaths), Argentina (12^th^ in contagions and deaths), Peru (18^th^ in contagions and 14^th^ in deaths), Chile (25^th^ in contagions and 21^st^ in deaths), and Ecuador (47^th^ in contagions and 24^th^ in deaths) [11, 12].

The mental health effects of previous or current SARS-Cov-2 pandemics [13, 14] are known, with healthcare workers being the best studied group due to the dangers arising from proximity to infected people and having to manage situations of stress and uncertainty [15]. Healthcare workers are at increased risk of developing the disease [16] and spreading it [17] due to their proximity while treating infected people. Early mental effects of the pandemic [18], with high levels of anxiety and depression [19–21], insomnia [15], emotional disorders [22], or post-traumatic stress disorder [23] have been found.

Studies in Latin America have found that 66% of respondents had had a deceased family member, friend, or acquaintance, with mental health effects affecting their degree of care, understanding, decision-making, and overall well-being [24, 25]. The vulnerable groups identified are women, young people, self-employed workers, and people with previous psychological processes with treatments that had been interrupted due to the pandemic [26]. The hypothesis is that the current pandemic generates effects on mental health, but with the need for studies to corroborate it [27], as has been found in studies conducted at the international level [28].

The objective of the study was to analyse the level of psychological distress in the population of Ecuador during the first phase of the COVID-19 pandemic, identifying the possible association with sociodemographic variables, presence of physical symptoms, and contact history in order to be able to establish preventive measures and find out whether the results differ from those found in other geographical areas.

## Materials and methods

### Design type and sample

Cross-sectional observational study. This investigation followed the STROBE guidelines.

The total number of questionnaires analysed was 3640, collected between 2 April and 17 May, 2020. The inclusion criteria were: being 18 years of age or older, residing in Ecuador during the pandemic, and accepting the informed consent. Questionnaires were received from the 24 provinces of Ecuador. A strict selection criterion was adopted, eliminating all questionnaires with a response rate of less than 99% (857 questionnaires out of 4497 received).

### Instruments

This study is integrated into a research coordinated from Spain which is carried out in a total of 16 countries, Latin American, European, African, and Asian, at different stages of adaptation and implementation, and using a similar methodology, except for the differences generated with the adaptation to each country or the dates of data collection. The original questionnaire has been validated for the Spanish population, adapting questions from previous studies [29] and reviewing literature on publications from previous epidemics [15]. To facilitate its validation and to not delay its process over time, because of the need to collect data on the effects at the onset of the pandemic, previously validated instruments were included. The draft questionnaire was analysed by a panel of experts consisting of psychologists, occupational physicians and nurses, epidemiologists, and public health experts. A pilot test was conducted involving 57 people from different professions, educational levels, sex, age, and geographic areas, and no understandability problems or relevant incidents were identified, with a Cronbach’s alpha coefficient of 0.86. Subsequently, the questionnaire was culturally adapted to the Ecuadorian population, modifying the questions that had difficulty of being understood by the citizens of the country.

The questionnaire includes sociodemographic data: sex, age, cohabiting people, level of studies, employment status, having children or not, pets, or a disability.

Psychological adjustment was measured using a widely used tool to assess mental health and psychological well-being: the Goldberg’s General Health questionnaire (GHQ-12) [30]. This questionnaire consists of 12 items with four answer options. The first two are assigned a score of 0 points and the last two are assigned a score of 1 point, with a total score ranging from 0 to 12. The set cut-off point for the general population was 3, considering psychological distress for those with scores greater than or equal to 3.

Data were also collected on perceived symptoms over the last 14 days: cough, headache, rhinitis, fever, myalgia, dizziness, sore throat, chills, diarrhoea, or shortness of breath. This was provided by the World Health Organization on the most common physical symptoms associated with COVID-19. The subjects were questioned about whether they had a chronic illness or if they were taking medication at the time of answering the questionnaire; similarly, if they had been hospitalised or had required medical care in the last 14 days.

They were also asked about contact history in the last 14 days, including three items: possible contact (more than 15 min less than two metres away); casual contact with confirmed infected persons; or contact with people or materials suspected of being infected, as well as the existence of an infected family member or co-worker diagnosed by diagnostic testing. Self-perceived health status was measured with five response levels, from lousy to optimal, grouping them for the final analysis into two categories, being this a well-known good indicator for predicting mortality [31].

Preventive measures were assessed through questions with five answer choices, categorised from never to always, regarding how often the following behaviours were identified: covering mouth with elbow when coughing or sneezing; avoiding sharing utensils (e.g. fork) during meals; washing hands with soap and water; washing hands with hydroalcoholic solution; washing hands immediately after coughing, touching the nose, or sneezing; washing hands after touching potentially contaminated objects; wearing a mask regardless of the presence of symptoms; leaving at least a metre and a half distance between others.

### Procedure

**Fig 1:**
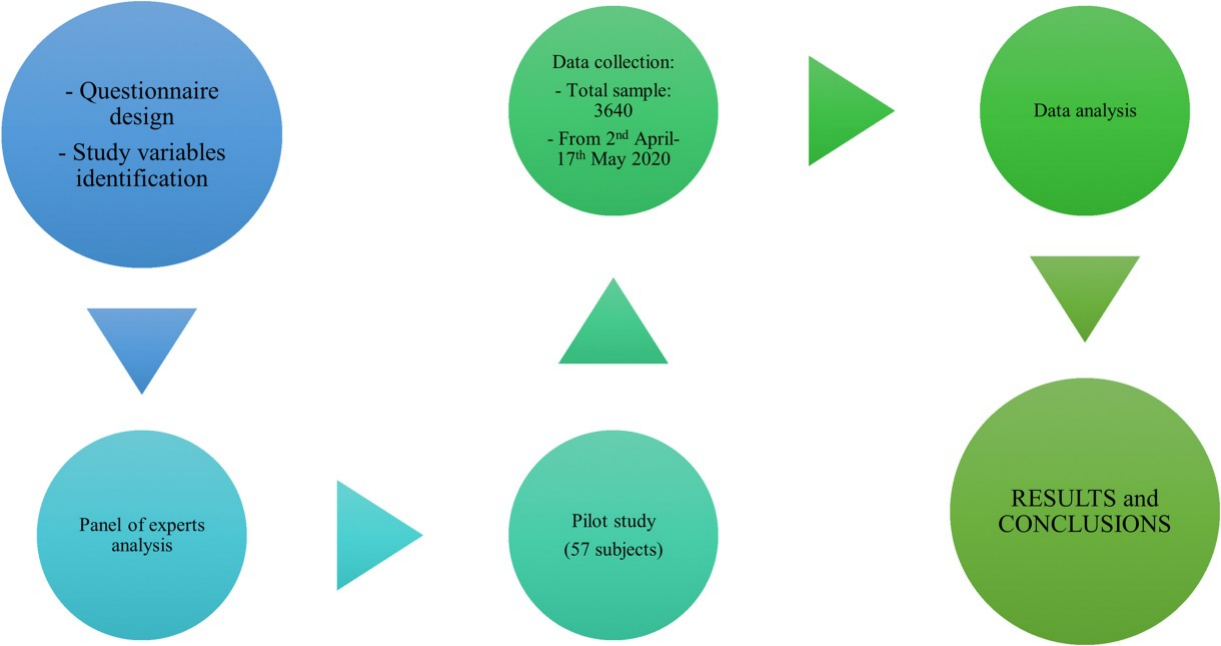
Study phases.

Data were collected through an online questionnaire, the Qualtrics storage and surveys platform®. In this way, the confinement measures established during the pandemic did not interfere with the data collection process. For sampling, the non-probabilistic sampling methodology was used: snowball methods, the same methodology chosen to carry out the study in Europe on Living, Working and COVID-19 by Eurofound [32]. Universities and scientific societies were involved in the process of disseminating information, as well as social media and the press. The questionnaires were collected in the first phase of the pandemic, between 2 April and 17 May 2020, with the health alert being decreed in Ecuador thirteen days before the start of the study (Fig 1).

### Data analysis

After cleaning-up the database, frequencies, mean, and standard deviation were determined based on the type of variable. The normality study of data distribution was carried out using the Kolmogorov-Smirnov test, obtaining a value of p>0.20. Therefore, the chi-squared association test and the Student’s T test were implemented for independent samples, which made it possible to contrast whether there was existence or not of a relationship between the different variables (sociodemographic, physical symptoms, self-perceived health status, history of contact with people infected with SARS-CoV2 virus or contaminated material, and preventive measures taken) with respect to the presence or not of psychological distress.

The binary logistic regression analysis identified among the studied variables those that played a more relevant role, and a model was built to determine whether psychological distress was present. The selection of variables was carried on forward, considering the likelihood ratio statistic. Odd Ratios (OR) were estimated, and confidence intervals were provided for this association measure. In addition, different goodness-of-fit measures were used: Hosmer-Lemeshow test, percentage of correctly classified values, sensitivity, and specificity.

All analyses were carried out with the SPSS 26.0 statistical software (IBM, Armonk, NY, USA).

### Ethical principles

The ethical principles set out in the Declaration of Helsinki have been followed. The participants’ permission was obtained through an informed consent in which they expressed their voluntary desire to participate in the study. At the beginning of the online survey, subjects had to indicate that they were of legal age and that they participated voluntarily in the study in order to access the content of the study. The data was recorded anonymously and treated confidentially. The study has been authorised in Ecuador by the Research Ethics Committee of the San Gregorio de Portoviejo University (USGP-DI-049-2021), and, in Spain, by the Research Ethics Committee of Huelva, belonging to the Regional Ministry of Health of Andalusia, Spain (PI 036/20).

## Results

### Sociodemographic data

The sample analysed, amounting to a total of 3640 subjects, is slightly higher for the female sex (54.92%), with an age at which the percentage at 30 years or less was of 52.81%, and a marital status in which 61.43% had no partner. As regards the educational level, 74.15% had upper secondary education or lower, and 25.85% had university studies or higher. In relation to their occupation, 45.07% were public employees, 39.84% worked in a private company, and 15.09% were self-employed. In the sample, the percentage of those who didn’t have children was lower (45.4%). A majority claimed to have a pet (58.27%), and only 2.36% had some kind of disability (Table 1).

**Table 1:** Association between sociodemographic variables and psychological distress during the pandemic.

		GHQ					
	N (%)	Yes	No	χ^2^		p	Odds Ratio (Confidence Interval at the 95 level)
	(N = 3640)	(N = 2283)	(N = 1357)				
**Sex**							.546
Male	1641 (45.1)	55.0	45.0	76.822		< .001	
							(.477, .626)
Female	1999 (54.9)	69.1	30.9				
Age*							1.119
30 years old or younger	1900 (52.8)	64.1	35.9	2.650		.104	
							(.977, 1.281)
Older than 30	1698 (47.2)	61.4	38.6				
**Marital status**							1.169
Without a partner	2236 (61.4)	64.1	35.9	4.947		.026	
							(1.019, 1.341)
With a partner	1404 (38.6)	60.5	39.5				
**Level of studies**							.779
Upper secondary school or lower	941 (25.9)	58.3	41.7	10.401		.001	
							(.679, .907)
University or higher	2699 (74.1)	64.2	35.8				
You are**							
Self-employed	326 (15.1)	61.0	39.0	5.760		.056	
Public worker	974 (45.1)	65.3	34.7				
Private-company worker	861 (39.8)	60.0	40.0				
**Children**							.804
Yes	1652 (45.4)	59.9	40.1	10.087		.001	
							(.702, .920)
No	1988 (54.6)	65.0	35.0				
**Pet**							1.011
Yes	2121 (58.3)	62.6	37.4	0.025		.874	
							(.882, 1.159)
No	1519 (41.7)	62.9	37.1				
**Disability**							.907
Yes	86 (2.4)	60.5	39.5	0.191		.662	(.586, 1.405)
No	3554 (97.6)	62.8	37.2				

*Grouped variable from the median value.

### Psychological distress in the sample

As can be seen, in Table 2, 62.72% of the sample has psychological distress (PD), with a cut-off point of GHQ ≥ 3. The overall score on the 12 items is M = 4.41 (SD = 3.49).

**Table 2:** Psychological distress: General health questionnaire GHQ-12.

	TOTAL (N = 3640)
Item	M (SD)
1. Have you been able to properly concentrate on what you were doing?	2.44 (0.76)
2. Have your worries made you lose a lot of sleep?	2.57 (1.00)
3. Have you felt you are developing a relevant role in life?	2.00 (0.88)
4. Have you felt capable of making decisions?	1.99 (0.78)
5. Have you felt constantly overwhelmed and stressed?	2.66 (0.94)
6. Have you felt unable to overcome your difficulties?	2.18 (0.96)
7. Have you been able to develop your normal daily activities?	2.62 (0.92)
8. Have you been able to properly face your difficulties?	2.23 (0.75)
9. Have you felt unhappy or depressed?	2.36 (0.98)
10. Have you lost confidence in yourself?	1.74 (0.92)
11. Have you thought that you are a worthless person?	1.39 (0.78)
12. Do you feel reasonably happy given the circumstances?	2.13 (0.79)
GHQ-12 (Score on a scale of 12)	4.41 (3.49)
**Cut-off point ≥ 3**	**N (%)**
Yes	2283 (62.72)
No	1357 (37.28)

Cronbach’s α = 0.815.

The three items with the highest rating (M>2.5) have been items 5: Have you felt constantly overwhelmed and stressed? M = 2.66 (SD = 0.94); item 7: Have you been able to develop your normal daily activities? M = 2.62 (SD = 0.92); and item 2: Have your worries made you lose a lot of sleep? M = 2.57 (SD = 1.00). In contrast, items with a lower rating (M<2 or less than 2) have been items 11: Have you thought that you are a worthless person? M = 1.39 (SD = 0.78); item 10: Have you lost confidence in yourself? M = 1.74 (SD = 0.92); and item 4: Have you felt capable of making decisions? M = 1.99 (SD = 0.78) (Table 2).

### Sociodemographic data and psychological distress

Women report a higher percentage of psychological distress (69.1%) than men (55.0%), p < .001, OR = 0.546, 95% CI = (0.477, 0.626). People without a partner have higher PD (64.1%) than those who have a partner (60.5%), p = .026, OR = 1.169, 95% CI = (1.019, 1.341). Those with university studies show a higher percentage of PD (64.2%) than those with a lower level of studies (58.3%), p < .001, OR = 0.779, 95% CI = (0.679, 0.907). Not having children is associated with a higher percentage of PD, 65.0% vs. 59.9%, p < .001, OR = 0.804, 95% CI = (0.702, 0.920) (Table 1).

There are no statistically significant differences in PD regarding age, type of employment (public, private, or self-employed), having a pet, or having a disability (Table 1).

### Physical symptoms, health-related variables and psychological distress

Among the most common symptoms in the 14 days prior to the participation in the study (Table 3) the ones with a higher percentage of cases are headache (40.80%), coryza (24.12%), and sore throat (22.83%). In contrast, less frequent symptoms have been fever (> 38°C for at least 1 day) 4.42%, chills (4.48%), and breathing difficulty (4.78%). The set of symptoms, with an M = 1.59 (SD = 1.86), is associated with the level of PD, M = 1.86 (SD = 1.96) for those with PD versus M = 1.14 (SD = 1.56) for those without PD, p < .001.

**Table 3:** Association between physical symptoms, current health status, history of contacts, and psychological distress during the pandemic.

		GHQ				
	N (%)	Yes	No	χ^2^	p	Odds Ratio (Confidence Interval = 95)
		(N = 2283)	(N = 1357)			
**PHYSICAL SYMPTOMS**						
**Fever**						
Yes	161 (4.4)	75.2	24.8	11.141	.001	1.843
No	3479 (95.6)	62.1	37.9			(1.281, 2.652)
**Cough**						
Yes	623 (17.1)	70.5	29.5	19.287	< .001	1.518
No	3017 (82.9)	61.1	38.9			(1.259, 1.830)
**Myalgia**						
Yes	1485 (40.8)	72.7	27.3	107.436	< .001	2.110
No	2155 (59.2)	55.8	44.2			(1.830, 2.433)
**Muscle pain**						
Yes	709 (19.5)	74.8	25.2	54.529	< .001	1.990
No	2931 (80.5)	59.8	40.2			(1.654, 2.394)
**Dizziness**						
Yes	378 (10.4)	79.6	20.4	51.583	< .001	2.525
No	3262 (89.6)	60.8	39.2			(1.947, 3.274)
**Diarrhoea**						
Yes	388 (10.7)	75.5	24.5	30.411	< .001	1.956
No	3252 (89.3)	61.2	38.8			(1.536, 2.491)
**Sore throat**						
Yes	831 (22.8)	73.5	26.5	53.778	< .001	1.889
No	2809 (77.2)	59.5	40.5			(1.591, 2.242)
**Rhinitis**						
Yes	878 (24.1)	69.7	30.3	24.138	< .001	1.502
No	2762 (75.9)	60.5	39.5			(1.276, 1.768)
**Chills**						
Yes	163 (4.5)	71.8	28.2	5.990	.014	1.539
No	3477 (95.5)	62.3	37.7			(1.087, 2.180)
**Shortness of breath**						
Yes	174 (4.8)	76.4	23.6	14.705	< .001	1.986
No	3466 (95.2)	62.0	38.0			(1.390, 2.837)
**CURRENT HEALTH STATUS**						
**Self-perceived health**						.431
Optimal	3039 (83.5)	59.8	40.2	67.596	< .001	
Mediocre or lousy	601 (16.5)	77.5	22.5			(.351, .529)
**Chronic illness**						1.177
Yes	560 (15.4)	65.9	34.1	2.850	.091	
No	3080 (84.6)	62.1	37.9			(.974, 1.422)
**Currently taking medication**						1.318
Yes	696 (19.1)	67.8	32.2	9.559	.002	
No	2944 (80.9)	61.5	38.5			(1.106, 1.571)
**Admitted to hosp. Last 14 days**						1.340
Yes	26 (0.7)	69.2	30.8	.475	.491	
No	3614 (99.3)	62.7	37.3			(.581, 3.090)
**Medical care last 14 days**						1.502
Yes	222 (6.1)	71.2	28.8	7.222	.007	
No	3418 (93.9)	62.2	37.8			(1.114, 2.025)
**CONTACT HISTORY**						
**Contact >15’ <2m with infected person**						1.504
Yes, or doesn’t know	1351 (37.1)	68.6	31.4	31.941	< .001	
No	2289 (62.9)	59.2	40.8			(1.305, 1.734)
**Casual contact with infected person**						1.487
Yes, or doesn’t know	1307 (35.9)	68.6	31.4	29.685	< .001	
No	2333 (64.1)	59.5	40.5			(1.289, 1.716)
**Contact with person or material suspected of being infected**						1.407
Yes, or doesn’t know	1596 (43.8)	67.2	32.8	24.051	< .001	
No	2044 (56.4)	59.2	40.8			(1.227, 1.613)
**Infected family member**						1.353
Yes, or doesn’t know	886 (24.3)	67.9	32.1	13.678	< .001	
						(1.152, 1.589)
No	2754 (75.7)	61.0	39.0			
**Has been performed diagnostic test**						.886
Yes	336 (9.2)	60.1	39.9	1.071	.301	
						(.704, 1.114)
No	3304 (90.8)	63.0	37.0			

There is a statistically significant difference between having any of the studied symptoms or not and presenting PD, as can be seen in Table 3. The symptoms with a higher percentage that are found among those with PD are: dizziness 79.6%, OR = 2.52, 95% CI = (1.947, 3.274); breathing difficulty 76.4%, OR = 1.98, 95% CI = (1.390, 2.837); diarrhoea 75.5%, OR = 1.96, 95% CI = (1.536, 2.491); fever 75.2%, OR = 1.84, 95% CI = (1.281, 2.652); myalgia 74.8%, OR = 1.99, 95% CI = (1.654, 2.394); and sore throat 73.5%, OR = 1.89, 95% CI = (1.591, 2.242) (Table 3).

The number of symptoms, with a rating of M = 1.59 (SD = 1.86), is different for those with high PD, M = 1.86 (SD = 1.96) and those with low PD, M = 1.14 (SD = 1.56), p < .001.

83.49% of participants stated an optimal self-perceived health, relating it to the level of PD. Thus, among those with optimal health, 59.8% had PD, a percentage that increases to 77.5% among those who had mediocre or lousy health, p < .001, OR = 0.431, 95% CI = (.351, .529). 19.12% were taking medications, who also had a higher percentage of people with PD, 67.8%, than those who did not take medication, 61.5%, p.002, OR = 1.32, 95% CI = (1.106, 1.571). 6.10% had received medical care over the past 14 days, associated with developing PD; thus, among those who had received medical care in the last 14 days, 71.2% had PD versus 62.2% who had not received it, p.007, OR = 1.50, 95% CI = (1.114, 2.025). 15.38% had a chronic disease, and 0.71% had required hospitalisation in that time period, without having found a statistically significant association between these two variables and developing PD (Table 3).

### Contact history and psychological distress

37.1% of the sample knew they had had contact with an infected person for more than 15 minutes and/or within less than 2 metres distance or did not know if they had, compared to 62.9% who claimed to not have been in such contact. The percentage with PD among those who had been in contact, or did not know, was greater (68.6%) than among those who had not (59.2%), p<,001, OR = 1.50, 95 CI = (1.305, 1.734) (Table 3).

The percentage of those who had had casual contact with an infected person or did not know if they had (35.9%) was lower than those who claimed to not have been in such contact (64.1%), with higher percentage of PD among those who had had contact (68.6%) than those who had not (59.5%), p < .001, OR = 1.48, 95% CI = 1.289, 1.716). The percentage of those who had had any contact with a person or material suspected of being infected, or did not know if they had, (43.8%) was lower than those who claimed they had not been in such situation (56.4%), with the highest percentage of PD among those who had had contact, p < .001, OR = 1.41, 95% CI = (1.227, 1.613). Similarly, the percentage of those who had had contact with an infected family member or did not know if they had (24.3%) was lower than those who had not (75.7%), p < .001, OR = 1.35, 95% CI = (1.152, 1.589) (Table 3).

9.2% had had a diagnostic test, with no statistically significant association between having PD and having been performed the diagnostic test (Table 3).

### Preventive measures and psychological distress

As can be seen in Table 4, the highest scoring preventive measure is "Washing hands with soap and water" M = 4.73 (SD = 0.55), followed by "Washing hands after touching potentially contaminated objects" M = 4.65 (SD = 0.69). Not too far away and with similar values, there are preventive measures such as: "Wearing a mask regardless of the presence of symptoms" M = 4.55 (SD = 0.88), "Covering the mouth" M = 4.52 (SD = 0.77), and "Keeping at least a metre and a half distance" M = 4.51 (SD = 0.75). On the contrary, the least used preventive measures are: "Washing hands with hydroalcoholic solution" M = 4.20 (SD = 0.99), “Washing hands after coughing, touching the nose or sneezing" M = 4.14 (SD = 1.01), and "Avoiding sharing utensils" M = 4.14 (SD = 1.23).

**Table 4:** Contrast between preventive measures and psychological distress during the pandemic.

	TOTAL (N = 3640)				
		GHQ			
	M (SD)	Yes	No	Statistical	p
**Covering mouth**	4.52 (0.77)	4.49 (0.80)	4.58 (0.72)	-3.244	.001
**Avoiding sharing utensils**	4.14 (1.23)	4.08 (1.24)	4.24 (1.19)	-3.774	< .001
**Washing hands with soap and water**	4.73 (0.55)	4.72 (0.57)	4.76 (0.52)	-2.359	0.018
**Washing hands with hydroalcoholic solution**	4.20 (0.99)	4.18 (0.99)	4.24 (0.99)	-1.817	.069
**Washing hands immediately after coughing, touching the nose, or sneezing**	4.14 (1.01)	4.10 (1.02)	4.20 (0.98)	-2.918	.004
**Washing hands after touching potentially contaminated objects**	4.65 (0.69)	4.63 (0.69)	4.68 (0.67)	-1.906	.057
**Wearing a mask regardless of the presence of symptoms**	4.55 (0.88)	4.52 (0.89)	4.59 (0.87)	-2.062	.039
**Leaving at least a metre and a half distance between others**	4.51 (0.75)	4.50 (0.74)	4.54 (0.77)	-1.475	0.140

Note: Likert-type answer scale from 1 (Never) to 5 (Always).

A statistically significant association has been found between having PD and using the following preventive measures: “Covering the mouth”, “Avoiding sharing utensils”, “Washing hands with soap and water”, “Washing hands after coughing, touching the nose, or sneezing”, and “Wearing a mask regardless of the presence of symptoms” (Table 4).

### Prediction of psychological distress during the pandemic

Psychological stress during the pandemic in Ecuador is predicted by the variables: being female OR = 1.765, 95% CI = (1.536, 2.028); having university studies OR = 1.284, 95% CI (1.098, 1.501); not having children OR = 1.285, 95% CI = (1.118, 1.477); poor self-perceived health over the past 14 days OR = 1.628, 95% CI = (1.302, 2.037); and a higher number of symptoms OR = 1.209, 95% CI = (1.154, 1.266). These variables predict 65.5%, with a sensitivity/specificity of 25.6 / 88.9, R^2^ = 0.063. The results of the Hosmer-Lemeshov Test are χ^2^ = 14.629 (p = 0.067) and of the omnibus test, χ^2^ = 238.050 (p<0.001).

## Discussion

This study allows to know the effects of the SARS-Cov-2 pandemic on the mental health of the Ecuadorian population, in particular at the level of psychological distress (PD) presented in the first phase of the pandemic. It has been assessed how various sociodemographic factors, physical symptoms, history of contact with the virus, and preventive measures intervene in the development of PD. It was found that the results differ from those obtained in other countries, which makes it easier to identify those factors that, after working on them, can help minimise the negative effects of the pandemic for future outbreaks or in future epidemics.

It has been suggested that the limited economic support measures for the most disadvantaged groups, in much of Latin America, must have influenced the effects of COVID-19 on the citizens of these countries [33], and that socio-cultural factors should also be taken into account when analysing the effects of the legislative measures taken by the governments of those countries to combat the pandemic [11]. These are seen as aspects that may explain the differences in study results in Ecuador from those found in countries outside Latin America. A percentage with high psychological distress (GHQ-12: cut-off point ≥ 3) has been lower in Ecuador (62.72%) than the one found in Spain (72.0%) [34], with a total scale score (over 12 points) in Ecuador of M = 4.41 (SD = 3.49) versus M = 4.99 (SD = 3.34). These differences may be explained because, in the Ecuadorian sample, the percentage of women and participants with university studies was lower than the one found in the study carried out in Spain, being both situations (higher level of studies and being female) associated with a higher level of PD, a fact found in studies conducted both in Europe [28] and Latin America [26], with the exception of Chile, where men showed higher psychological distress than women [35]. A study in 28 European countries found that men are more optimistic than women about the pandemic and that this difference increased over time [32]. Precisely, this optimism can be related to optimal self-perceived health, as we can see when observing in the present study that an optimal perception is associated with lower PD, something that is not uncommon since it is well known that health perception is a good predictor of mortality (31). Women in Ecuador do not perceive their health as optimal in a higher percentage, statistically significant, than men (54.3% vs. 45.7%, p.074). Global statistics give a higher percentage of COVID-19 contagion lethality among men, but the World Bank says coronavirus is not gender-blind and that women are most affected by the pandemic in terms of health risks, pre-existing job inequalities, and responsibilities to care for others [36], which corresponds to the higher percentage of PD in the women of the study at hand.

Younger people have had their mental health more affected, as well as those who do not live with children and children under the age of 16. The fact that young people have been more affected has been seen in previous studies in Ecuador [35], Spain [35, 37], and Colombia [35], although in Chile the elderly were the most affected [35]. It may seem that living with children or young people generates more work, because of care needs, but this is compensated by the positive influence their company has on mental health, something that has not been seen when having a pet. Likewise, it can be understood that young people suffer from the effects of confinement and movement restriction to reduce contagion in a higher degree. Also, having any degree of disability increases PD, explainable by the low percentage, 2.4% (86 cases), that claimed to have it.

In this study, the most commonly found symptoms were: headache, sore throat, and nasal congestion, while the European study detected two other symptoms in second and third place: cough, and myalgia [34]. With regard to preventive measures to prevent contagion, three have been found with clearly greater use compared to the study carried out in Spain [28]: "Wearing a mask regardless of the presence of symptoms", "Washing hands with hydroalcoholic solution", and "Washing hands after coughing, touching the nose, or sneezing". The social customs or information received may be behind these differences, considering that the data in Ecuador were obtained in a period of time later than in Spain. For example, the use of a mask was not recommended by the WHO at the general level, nor did governments force its use at the earliest moments of the pandemic.

The percentage that claimed that no family member was infected (75.7%) was much lower than that found in previous studies in Spain (97.8%), and the percentage of those who had had a diagnostic test in the previous 14 days (9.2%) was higher than the one found at European level (6.1%) [34]. An explanation of this difference, apart from the socio-economic level and the health system, may be the different dates for data collection or the type of diagnostic test performed.

In a study conducted in the 28 countries of Europe in two phases of the pandemic, at the beginning and after 6 months of its epidemiological evolution, it has been observed that the countries most affected by the health crisis (France, Italy, and Spain) were among those with the greatest improvement in mental well-being between April and July. The study has shown that optimism had increased more in countries that had established pandemic movement restrictions than among those that had not [32], contradicting the influence of confinement on mental health [38].

In Ecuador, the ethnicity variable is of great importance because of the number and the diversity of ethnicities existing in the country. It is known to be a variable that influences inequality with respect to healthcare services [39], so it can be inferred that it will have influenced the effects of COVID-19 on mental health during the pandemic. In the study at hand, the ethnicity variable was not contemplated, and no differences have been observed in PD either by province or by type of housing.

Having obtained data about the COVID-19 effects in the first phase of the pandemic in Ecuador will allow to complete it in a second phase and thus, know the effect differences on mental health. Moreover, as proposed by Thakur and Jain [40], it is necessary to assess the emotional distress of the population in relation to COVID-19 in order to proceed with the design of intervention strategies for the improvement of mental and public health. Therefore, this study is a first step towards understanding the impact of the pandemic in Ecuador, and its results could help the Ecuadorian authorities in the design of actions to mitigate the emotional impact of the pandemic on their population. Future research should include specific analysis of variables such as economy, culture, ethnicity, or availability of healthcare resources while studying treatment and vaccines, as well as other traditional medicine approaches, to control the pandemic [41].

On the other hand, there are new factors, still subject to effectiveness studies, that could contribute to ameliorating the mental health impact of the COVID-19 pandemic. For example, a high-fibre, plant-based diet, which happens to be consumed by the majority of the Indian population, appears to be advantageous as it confers several health benefits on the host, including enhanced immunity to the COVID-19 disease [42].

In this context, new instruments that measure fear and anxiety towards COVID-19 have been proposed, such as the short questionnaire validated in the Spanish population [43], which facilitate the rapid and reliable evaluation of the presence of anxiety and fear of COVID-19, and thus allow to obtain results in order to implement public health improvement plans.

The limitations of this study are related to the non-probabilistic sampling technique used, as well as the inequality between the percentages of participating men and women (69% of the sample was composed by women), which could affect results from a gender perspective. This issue should be considered in future studies with the aim of obtaining more significant results from a gender perspective. Also, these results should be considered with caution due to the cross-sectional design of this investigation.

## Conclusions

The variables that best predict psychological distress in our study were being a woman, not living with children or children under the age of 16, having university studies, perception of the health status, and overall number of symptoms.

It has also been found that young people have had their mental health more affected, foreseeably by worse management of confinement measures or by other variables such as: living without a partner, taking medication, or requiring recent medical care.

On the contrary, among the variables that have not influenced the level of PD, there are: working in a public/private company or being self-employed, having a pet, or having some degree of disability.

The three most common symptoms have been: headache, sore throat, and nasal congestion, and the three preventive measures to prevent contagion: "Wearing a mask regardless of the presence of symptoms", "Washing hands with hydroalcoholic solution", and "Washing hands after coughing, touching the nose, or sneezing”.

Differences have been observed, as compared to other European studies, in the lowest overall percentage of people with psychological distress, the type of most common symptoms, preventive measures taken to prevent contagion, infected relatives, or percentage of those who have been performed a diagnostic test. This can be justified by differences in sociodemographic variables, the moment of the pandemic when data were collected, cultural characteristics, health system, or level of received information.

The use of the same country-adapted research instrument has facilitated the comparison of results, even if large economic and socio-cultural differences between countries are maintained. A second study, to be carried out in an advanced phase of the pandemic, will allow to know the differences experienced in the mental health of Ecuadorians between the first phase and after many months of the pandemic.

## Supporting information

S1 Checklist STROBE statement—Checklist of items that should be included in reports of *cross-sectional studies*. (DOCX)
